# The validity and reliability of counter movement jump height measured with the Polar Vantage V2 sports watch

**DOI:** 10.3389/fspor.2022.1013360

**Published:** 2022-10-28

**Authors:** Markus Gruber, Jussi Peltonen, Julia Bartsch, Philipp Barzyk

**Affiliations:** ^1^Human Performance Research Centre, Department of Sport Science, University of Konstanz, Konstanz, Germany; ^2^Polar Electro Oy, Kempele, Finland

**Keywords:** vertical jump, IMU, flight time, neuromuscular power, leg extension, performance, fitness test

## Abstract

The purpose of the present study was to assess the validity and reliability of the jump height measured by the Polar Vantage V2 sports watch in comparison to a gold-standard force plate measurement. Fifteen healthy adults, seven female, age 20–42 years participated in the study and performed six sets of three CMJs, on two consecutive days. The participants wore the Polar Vantage V2 sports watch (Polar Electro Oy, Kempele, Finland) whilst performing the jumps on two force plates (AMTI, Watertown, Massachusetts, United States). Jump height was on the one hand extracted directly from the watch (“leg recovery test”) and on the other hand calculated by the flight time method with the force plate data. To assess validity, we calculated the mean absolute error, constructed Bland-Altman plots and applied an ordinary least squares regression analysis. To test for left-to-right and day-to-day reliability, we calculated Pearson and intraclass correlations. We found a mean error of ≈5% and a high correlation (r = 0.96; *p* < 0.001) for the jump height measured by the Polar Vantage V2 sports watch compared to the force plate measurement. The Bland-Altmann plot together with the ordinary least squares regression analysis showed no systematic bias between the methods with a minimal difference at a jump height of 30 cm. For reliability of left-to-right and day-to-day measurements, we found high Pearson and ICC correlations and no indications for systematic bias by Bland-Altmann analysis. The present study has demonstrated that the “leg recovery test” of the Polar Vantage V2 sports watch provide a valid and reliable measurement of the mean vertical jump height of three consecutive CMJs. For the first time the jump height of a CMJ can be measured solely by a sports watch without the need to attach additional sensors or measurement devices. Thus, the “leg recovery test” is an easy to administer, valid and reliable test, that can be used in future studies to measure CMJ-height in the field when lab-based assessments are unavailable or inconvenient. This opens new avenues for cross-sectional and longitudinal assessments of neuromuscular power of the lower extremities in a large number of participants.

## Introduction

The countermovement jump (CMJ), which can be considered as the natural way for human beings to jump as high as possible without a run-up, has been extensively used to test neuromuscular performance in humans (for review see (1)). As jump height is a direct result of the generated impulse during leg extension, CMJ height is not only a task-specific measure of performance but reflects well the overall functional status of an individual (1). This is underlined by the fact that peak power during a CMJ is normally produced with jumping at bodyweight and neither loading nor unloading the body can increase peak power during the movement substantially (2). This optimum loading principle emphasize the functional importance of the CMJ as a test able to determine neuromuscular performance for daily life activities, like running, walking stairs, or simply standing up from a chair or bed (3). In this regard, CMJs have been successfully used to cross-sectionally assess neuromuscular performance in children (4), young adults (5) and elderly (6) and are further expected to be an effective type of exercise for sedentary people (7) as well as to monitor longitudinal effects of exercise [for review see (8)].

The gold standard to measure different outcome parameters of CMJs is a force plate measurement. As all forces that act at the bodies center of mass during the jump can be measured *via* the ground-reaction force, the jump height can easily be calculated from the force data. Jump height can be calculated *via* the flight time (time of zero force) with the classical law of ballistics, or it can be calculated by integration of the measured force over time during the jump (impulse method). Both methods have been shown to be valid (9), however, special care should be taken when directly comparing jump heights measured by the two distinct methods. Usually, the initial position for participants during the test is an upright stance with the entire foot on the ground. When jumping from this position the center of mass displacement derived by the impulse method is the difference of the center of mass during flat stance and the maximum height during the jump whereas for the flight time method the flight phase starts obviously at take-off position with a more extended ankle joint. Thus, jump heights that are calculated by the flight time method are obviously lower when directly compared to the impulse method (10).

For obvious reasons the calculation of jump height by impulse is restricted to force plate measurements whereas flight time can be detected by simpler measurement techniques. Recently, sensor-technology developed rapidly and a variety of systems have been brought to market maturity and have also been validated scientifically (for review see (11)). In principle two different categories of measurement methods can be distinguished. The first category comprises devices that measure the contact to the ground, like contact mats (12), light-barrier-systems (13) or video systems (14). The second category comprises inertial measurement units (IMUs) which are usually composed of accelerometers to detect linear acceleration and gyroscopes to measure rotational rate (see **Table 4**). Whereas in the first category the contact of the feet to the ground is measured with a device that is placed stationary outside the moving body (contact mat, light-barrier-system, camera), IMUs are attached to the body to capture the jump directly. In this regard IMUs provide the most feasible way to measure jump height outside the lab and in a large number of people. This is of utmost importance to obtain benefit from the CMJ-test in population studies and in broadly disseminating the test for the assessment of neuromuscular performance by practitioners (15).

The advantage of the measuring device being attached to the participants' body comes with the disadvantage that the attachment site of the IMU has to be chosen carefully. To the best of our knowledge only IMUs attached to the foot or upper/lower back (see **Table 4**) have been validated so far. The reported biases, random errors and correlations with jump heights calculated *via* force plate data in these studies (14, 16–20) show that IMUs in principle can be used to measure the jump height during a CMJ. Recently, Polar© integrated the “leg recovery test” into their Vantage V2 sports watch. The test consists of three CMJs and jump height is measured by an IMU, that is located inside the watch. To our knowledge, for the first time the jump height can be measured by a sports watch without the need to attach an additional sensor to the body. Thus, the test is technically and economically feasible to be used in a large population outside the lab in a real-world environment. The purpose of the present study was to assess the validity and reliability of the jump height measured by the Polar Vantage V2 sports watch in healthy adults.

## Methods

### Participants

Fifteen healthy adults (8 men and 7 women) aged 20 to 42 years (M = 26.4, ±5.6 years) took part in the study at the Human Performance Research Centre (HPRC) of the University of Konstanz. The study protocol was in accordance with the Declaration of Helsinki for human experimentation and the ethics standards of the University of Konstanz. The subjects were informed of the experimental risks and data processing procedures and signed an informed consent document prior to participation. Participants with acute severe injuries or with an injury in the last 6 months were excluded from the study (21).

### Study design

The participants performed two test sessions on subsequent days. On both days they first performed a standardized warm-up consisting of 20 jumping jacks, 10 squats and 10 sub-maximal CMJs. Afterwards, they performed three sets of three CMJs on two force plates (AMTI OR-6, Watertown, Massachusetts, United States), while wearing the Polar Vantage V2 sports watch (Polar Electro Oy, Kempele, Finland) and recording the “leg recovery test” which is integrated in the sports watch. Each set was separated by a 3-mins rest period and the rest period between individual jumps was 1 min. One set was performed with the Polar Vantage V2 at the right wrist, one set with the watch at the left wrist and one set with both watches simultaneously worn at the left and the right wrist. We used two different watches for the measurements, one watch was defined in the settings as a watch for the left wrist and another one was defined as a watch for the right wrist. The following day, the same watches were used for the same sides. In between subjects the order of sets was randomized.

The Polar Vantage V2 sports watch calculated the flight time of the CMJ by identifying the time between the take-off and landing by means of analysing the IMU data and then converting it to a jump height using the equation below where h is the jump height in meters, t is the flight time of the jump in seconds and g (9.81 ms^−2^) is the gravitational acceleration (22).


$$\begin{array}{l} h\;=\;\frac{1}{2}{\left(\frac{t}{2}\right)}^{2}g \end{array}$$

The same equation was used to calculate jump heights from the force plate data using an in-house written MATLAB (Version R2022a, The MathWorks, Inc., Natick, Massachusetts, United States) script.

In addition, we calculated jump height with the impulse method using an in-house written MATLAB script. We calculated the impulse (J) as the integral of the force (F) over the time interval from standing still on the force plate (t1) to take off (t2) which includes the whole countermovement (downward and upward phases of the jump). We used the trapezoidal rule for approximating the definite integral.


$$\begin{array}{l} J\;=\;\int_{t1}^{t2}F\;dt \end{array}$$

Then, we calculated the take-off velocity (v) using the following formula, with m being the participant's mass:


$$\begin{array}{l} v\;=\;\frac{m}{J} \end{array}$$

We calculated the jump height (h) with the following formula with v as the take-off velocity and g (9.81 ms-2) the gravitational acceleration.


$$\begin{array}{l} h\;=\;\frac{v^{2}}{2g} \end{array}$$

We included this analysis as supplementary data (Supplementary Figure A, Table A).

### Material

#### Sports watch

The Polar Vantage V2 sports watch (Polar Electro Oy, Kempele, Finland) integrates the measurement of jump height within the “leg recovery test.” The sports watch is worn at the wrist and uses the data of IMUs that are placed inside the watch to calculate the jump height of a CMJ. We used the instructions given by Polar: a) to prepare for the jump by placing hands firmly on hips and standing straight, b) to squat quickly and jump as high as possible by supplying power equally from both legs, c) not to bend the knees in the air before touchdown, d) to bend the knees after touchdown to allow smooth landing, e) to keep hands on hips throughout the entire movement (23). The watch displays the jump height for each individual jump in cm and the mean of the three jumps in cm without decimal places. We extracted the mean of the three jumps (one set) from the watch and used it for further analysis.

#### Force plate

The force plates (AMTI OR-6, Advanced Mechanical Technology Inc., Waterton, Massachusetts, United States) recorded the data at a sampling frequency of 1,000 Hz and were used to measure the flight time of the CMJs at the same time as they were recorded on the Polar Vantage V2 sports watch. They were connected to a computer equipped with the analysis software Vicon Nexus 2.10 (Vicon Motion Systems Ltd., Yarnton, United Kingdom). We defined the flight time between take-off and landing as the time during which the force was equal or <30 N. This time was then taken to calculate the jump height using the equation as described above (22).

In addition, we calculated the impulse and derived the jump height with the impulse method (see Bland-Altman Plots Supplementary Figure A, Table A).

#### Statistical analysis

We processed and analyzed all data with Excel (Version 16.53), MATLAB (Version R2022a) and JASP (Version 0.14.1) and calculated mean values, standard deviation (SD) as well as the coefficient of variation, as the ratio of the standard deviation (SD) to the mean, for all variables.

We calculated the mean absolute error (MAE) and the mean absolute percentage error (MAPE) with the following formulas, where yi is the jump height [ cm] calculated by the force plate data, xi is the jump height [ cm] calculated by the Polar Vantage V2 and n is the total number of measurements:


$$\begin{array}{l} MAE\;=\;\frac{1}{n}*\sum_{i\;=\;1}^{n}\left|y_{i}-x_{i}\right|\\ MAPE\;=\;\frac{100\%}{n}*\sum_{i\;=\;1}^{n}\left|\frac{y_{i}-x_{i}}{y_{i}}\right| \end{array}$$

In addition, we constructed Bland-Altman plots, to provide a representation of the agreement between the two methods (24) and applied an ordinary least squares (OLS) regression analysis to identify any systematic bias.

To test for reliability, we followed the recommendations of (25). We used Pearson's and intraclass correlation (ICC) to analyse left-to-right (jump height measured with the watch at the right wrist vs. jump height measured with the watch at the left wrist) and day-to-day reliability (jump heights measured during day 1 vs. jump heights measured during day 2). We considered Pearson's R scores of *r* > *0.8* (26) and ICC scores > *0.75* as high correlations (27).

## Results

**Figure 1:**
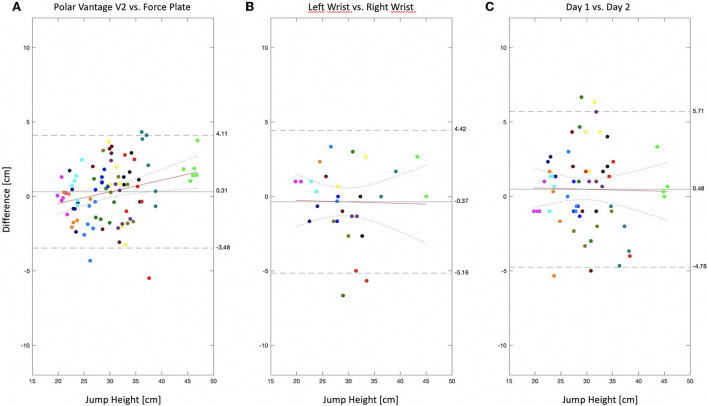
Bland-Altmann plots for **(A)** the difference of the jump height measured by the Polar Vantage V2 sports watch compared to the force plate measurement (jump height measured by force plate data—jump height measured by the Polar Vantage V2). **(B)** The left-right difference in CMJ-height when wearing one watch on the left and another watch on the right hand simultaneously (jump height measured by the Polar Vantage V2 on the left wrist – jump height measured by the Polar Vantage V2 on the right wrist) and **(C)** the day-to-day difference in CMJ-height (jump height measured by the Polar Vantage V2 on day 2 – jump height measured by the Polar Vantage V2 on day 1). The OLS regression lines are indicated as solid red lines with the 95% confidence intervals indicated as red dotted lines. The limits of agreement are marked as dashed black lines and given in numbers on the right side of the three plots together with the bias (mean of the differences) marked as solid black line. Same colored dots represent the mean of three jumps of one participant. Values are plotted over the CMJ-height derived *via* the flight time method from the force plate measurements.

The mean jump heights measured by the Polar Vantage 2 sports watch and the AMTI force plates were 29.93 cm (± 6.28 cm) and 30.24 cm (± 6.79 cm) respectively (Table 1). We were able to show a high correlation (r = 0.96; *p* < 0.001) between both methods and found a mean absolute percentage error of 5.19% for the jump height measured by the Polar Vantage V2 sports watch compared to the jump heights calculated by the force plate measurement. Figure 1A shows the comparison between the jump heights measured with the Polar Vantage V2 sports watch and the jump height measured with the force plates. In total, bias was 0.31 cm (higher jump height measured by the Polar Vantage V2). Please note that the slope of the regression line differed significantly from zero [*F*_(90)_ = 6.53; *p* = 0.01 with an R^2^ = 0.06] with the intersection point at 30 cm jump height, indicating that the difference between the jump heights derived from the force plate data and jump heights measured by the Polar Vantage V2 increase with jump heights that are lower or higher than 30 cm.

**Table 1:** Descriptive statistics and comparison between the CMJ-height measured by the Polar Vantage V2 sports watch and calculated *via* the flight time method from the force plate data.

	**Polar Vantage V2**	**Force plate**
Mean [cm]	29.93	30.24
Standard deviation [cm]	6.28	6.79
CV	0.21	0.22
Pearson's *r* [95% CI]	0.96^***^ [0.94,0.97]	
Mean absolute error [cm]	1.54	
Mean absolute percentage error [%]	5.19	

Level of significance: ^***^p < 0.001.

Results for the left-right reliability as well as for the test-retest (day-to-day) reliability are shown in Tables 2, 3. For the reliability analysis we only refer to jump heights measured by the Polar Vantage 2 sports watch. We found a high correlation between the jump heights measured while wearing the watch at the left wrist compared to wearing the watch at the right wrist respectively (r = 0.92, *p* < 0.001; ICC = 0.96, *p* < 0.001). For the day-to-day correlations, we found high scores (r = 0.91, *p* < 0.001; ICC = 0.79, *p* < 0.001) between the jump heights that were measured on consecutive days. In addition the regression analysis (Bland-Altmann Plots) showed no bias for either the left-to-right [*F*_(30)_ = 0.02; *p* = 0.90; R^2^ ≤ 0.001; Figure 1B] nor the day-to-day reliability [*F*_(60)_ = 0.03; *p* = 0.88; R^2^ ≤ 0.001; Figure 1C].

**Table 2:** Descriptive statistics and correlations of the left-right comparison between the CMJ-height measured by one Polar Vantage V2 sports watch attached to the right wrist and another one simultaneously attached to the left wrist.

	**Right wrist**	**Left wrist**
Mean [ cm]	29.57	29.20
Standard Deviation [ cm]	6.18	6.12
CV	0.21	0.21
Pearson's r [95% CI]	0.92^***^ [0.84,0.96]	
ICC [95% CI]	0.96^***^ [0.92,0.98]	

Level of significance: ^***^p < 0.001.

**Table 3:** Descriptive statistics and correlations of the day-to-day comparison between the CMJ-height measured by Polar Vantage V2 sports watch on two consecutive days.

	**Day 1**	**Day 2**
Mean [cm]	29.56	30.03
Standard deviation [cm]	6.31	6.25
CV	0.21	0.21
Pearson's r [95% CI]	0.91^***^ [0.85,0.95]	
ICC [95% CI]	0.79^***^ [0.73,0.84]	

Level of significance: ^***^p < 0.001.

## Discussion

The most important result of the present study is a rather small bias of 0.31 cm and mean absolute percentage error of 5.19% together with high correlations between CMJ-heights measured by the Polar Vantage V2 sports watch compared to the CMJ-heights measured *via* the force plates (see Figure 1A; Table 1). Error and bias are in the same range (17, 18, 20) or even lower (16, 19) compared to other studies that evaluated various IMU measurements against force plate derived CMJ-heights. Besides similarities in outcome, the biggest difference between the Polar Vantage V2 and the other devices, however, is the sensor placement (see Table 4). Whereas the IMU of the Polar Vantage V2 is located inside the sports watch itself, the IMUs of the other devices need to be attached to the body, usually foot or trunk. This comes with the big advantage that the test application is much simpler when using the Polar Vantage V2, but it comes with the disadvantage that you need to keep hands strictly attached to the hips during the CMJ. This difference has great implications for the potential field of application. Thinking of the assessment of jump heights during daily activities and sports, like e.g., the measurement of jump heights during a soccer game, the Polar Vantage V2 sports watch is inappropriate in its present condition. The preferred area of application of the Polar Vantage V2 can clearly be seen in the assessment of CMJ-heights in a broad population where the tests have to be conducted outside a laboratory with only minor or no involvement of experts and on a regular base. In this regard testing CMJ-height with the Polar Vantage V2 sports watch can be an appropriate measure to increase sample size in studies that aim to test neuromuscular performance cross-sectionally over the life-span (4–6) or longitudinally to test training interventions (7, 8). Recently, Dijkstra et al. (15) defined the term e-Sport-and-Exercise-Medicine (eSEM) as “the practice of SEM in athlete and public health contexts supported by electronic processes and communication.” Within this context a simple test like the CMJ can gain further importance for the assessment of neuromuscular performance (5, 28), which has been shown to act as a risk factor for frailty and other age related diseases (29, 30).

**Table 4:** Overview on studies that validated the measurement of CMJ-height by an IMU-device against CMJ-height calculated from force plate data.

**Study**	**IMU type**	**Placement**	**Correlation jump height IMU vs. Force plate (ICC or Pearson's r)**	**Error jump height IMU vs. Force plate (Random error, Bias)**
Garnacho-Castaño et al. (17)	Polar stride sensor (tri-axial accelerometer, sampling rate 100 Hz)	Sport shoe	ICC 0.97 (0.94–0.98 95% CI)	Bias−0.45 cm, Random error 1.85 cm
Choukou et al. (16)	Accelerometer (*Myotest*, sampling rate 500 Hz)	Middle of the lower back	ICC 0.79–0.86 (95% CI)	Bias 3.6 cm, random error 13.1 cm
Rantalainen et al. (19)	Accelerometer (sampling rate 100 Hz)	Back mid-line between scapulae at T1-T5 level	ICC 0.959 (95% CI)	Bias 4.3 cm
Pino-Ortega et al. (18)	WIMU (Realtrack, sampling rate 1,000 Hz)	Lower back on a belt	ICC 0.97 (0.96–0.98 95% CI)	Bias 0.29%
Setuain et al. (20)	IU (MTx, Xsens Technologies, sampling rate 100 Hz)	Middle of the lower back	Pearson's r 0.96 (0.89–0.99, *p* > 0.001)	Bias 1.96 cm

In line with previous studies that looked at comparing new non-IMU based measurement methods for jump height acquisition with force plate measurements, like the MyJump-App (14, 31) or the G-Flight micro-laser system (32), the results of the present study showed a slight overestimation of mean jump height at jump heights >30 cm but also an underestimation of jump heights below a jump height of 30 cm. Thus, the algorithm used to calculate the jump heights with the IMU data of the Polar sports watch shows its optimal performance at a jump height of 30 cm. It can be speculated that this is a result of the optimisation of the algorithm that is integrated in the Polar Vantage V2 at mean jump heights of an average study population, like the participants of the present study with an average jump height of 30 cm (Table 1). We were not able to find similar studies investigating jump height using wearables compared to force plates, that showed the same behavior. However, studies comparing jump-mats and motion capture systems showed similarly large differences between the methods at jump heights >0.30 cm (9, 33).

It has to be noted that the jump heights calculated *via* the flight time significantly differ from the jump height calculated based on the impulse derived from the measurement of the ground reaction force during the jump (see Supplementary material). The mean jump height calculated *via* the impulse method was 36.52 cm and thus 6.28 cm higher than the jump height calculated *via* the flight time method based on the force plate data and 6.59 cm higher than the jump height calculated by the Polar Vantage V2 sports watch (see Table 2; Supplementary material). This can be explained by a different definition of jump height between the methods and is well in line with the literature (10).

In a second part of the study, we checked the left-to-right and the day-to-day reliability of the CMJ-height measured by the Polar Vantage V2 sports watch. A low bias and high correlations (see Figure 1B; Table 2) between the jump heights of the simultaneous measurement of two watches, one at the left and one at the right wrist, indicated no systematic difference. Thus, the watch can be worn at either side without violating the measurement of CMJ-height. We obtained similar results, low bias and high correlations, also for the day-to-day reliability (see Figure 1C; Table 3). The day-to-day reliability provides the necessary precondition that can make the Polar Vantage V2 sports watch a valuable tool for longitudinal studies and a potential candidate within an eSEM strategy that aims to incorporate an outcome of neuromuscular performance.

The present study has several limitations, that should be considered. First and foremost, only fifteen participants took part in our study. Each of the participants performed six sets of three jumps each, resulting in six data points per participant (see Figure 1A). For the analyses we took the six means, which are not independent measures, into calculation, harbouring the risk to overvalue the correlations. Second, it has to be noted that the limits of agreement (see Figure1A) are still−3.48 cm and +4.11 cm respectively, meaning that the jump height for one person can differ by quite a large margin. We therefore argue that although the results may be valid for the mean of a large population, the results from an individual participant should be carefully considered. Third, we only tested healthy adults. A simple generalization of the results to groups of all age (children and seniors) and performance level (athletes) can therefore not be done easily.

## Conclusion

The “leg recovery test” of the Polar Vantage V2 sports watch can be used as a valid and reliable tool to assess the mean jump height of three successive CMJs. Without the need to attach any additional sensor to the body and given a good reliability over time and between sides, the sports watch provides an easy-to-perform test procedure that can be used to measure CMJ-height within a significant number of people. We suggest taking advantage of this technology to collect jump data from participants without the need for laboratory-based measurements thus enabling large-scale studies to be conducted at comparatively low costs.

## Data availability statement

The raw data supporting the conclusions of this article will be made available by the authors, without undue reservation.

## Ethics statement

Ethical review and approval was not required for the study on human participants in accordance with the local legislation and institutional requirements. The patients/participants provided their written informed consent to participate in this study.

## Author contributions

MG devised the project, designed the study, contributed to data analysis, and drafted the manuscript. JP contributed to the design of the study, helped with the data analysis, and reviewed the manuscript. JB helped with the experiments and data analysis and reviewed the manuscript. PB carried out the experiments, performed the computations and data analysis, and drafted parts of the manuscript. All authors contributed substantially to the article and approved the submitted version.

## Funding

The Polar Vantage V2 sport watches were provided by Polar free of charge for the measurements of the study.

## Conflict of interest

JP is employed as Senior Researcher at Polar Electro Oy, Kempele. The remaining authors declare that the research was conducted in the absence of any commercial or financial relationships that could be construed as a potential conflict of interest.

## Publisher's note

All claims expressed in this article are solely those of the authors and do not necessarily represent those of their affiliated organizations, or those of the publisher, the editors and the reviewers. Any product that may be evaluated in this article, or claim that may be made by its manufacturer, is not guaranteed or endorsed by the publisher.
