# Supplementary material for: The validity and reliability of counter movement jump height measured with the Polar Vantage V2 sports watch
Source: Front Sports Act Living. 2022 Oct 28;4:1013360. doi: 10.3389/fspor.2022.1013360 (PMC9650676; doi:10.3389/fspor.2022.1013360)
Supplement: Supplementary file 1 [file Table_1.DOCX]

Table A: Descriptive statistics and comparison between the jump heights calculated by the Polar Vantage V2 and the jump heights derived from the force plate data with the flight time method and the impulse method respectively.

|  | Mean [cm] | ±SD [cm] | CV |
| --- | --- | --- | --- |
| Polar Vantage V2 – Flight Time Method | 29.93 | 6.28 | .21 |
| Force Plate - Flight Time Method | 30.24 | 6.79 | .22 |
| Force Plate - Impulse Method | 36.52 | 7.38 | .20 |
