# Supplementary figures and images for: The validity and reliability of counter movement jump height measured with the Polar Vantage V2 sports watch
Source: Front Sports Act Living. 2022 Oct 28;4:1013360. doi: 10.3389/fspor.2022.1013360 (PMC9650676; doi:10.3389/fspor.2022.1013360)

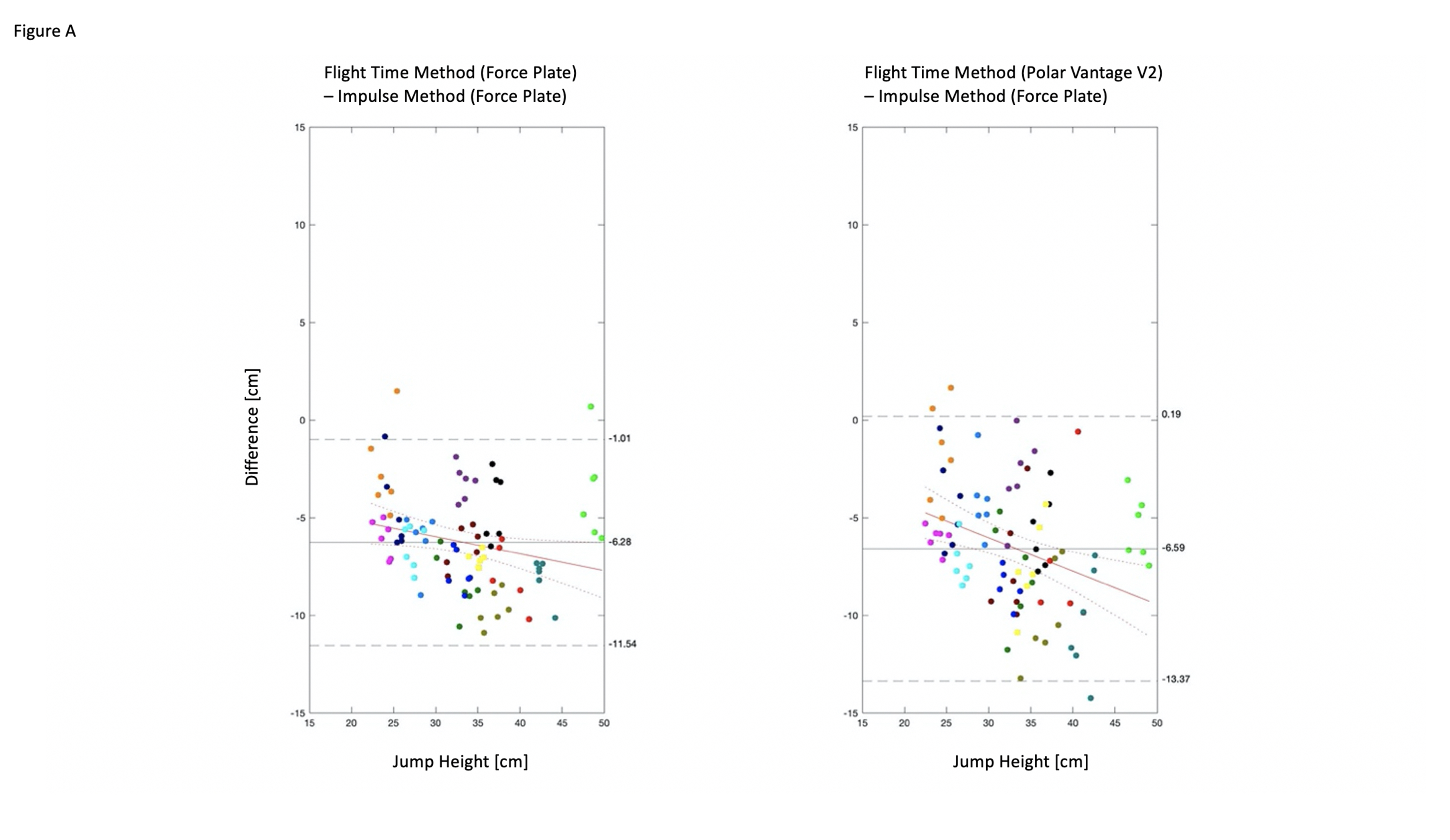

Supplement: Supplementary file 2 [file Image_1.JPG]
